# Activation of PPARγ suppresses proliferation and induces apoptosis of esophageal cancer cells by inhibiting TLR4-dependent MAPK pathway

**DOI:** 10.18632/oncotarget.10067

**Published:** 2016-06-15

**Authors:** Kai Wu, Yang Yang, Donglei Liu, Yu Qi, Chunyang Zhang, Jia Zhao, Song Zhao

**Affiliations:** ^1^ Department of Thoracic Surgery, The First Affiliated Hospital of Zhengzhou University, Zhengzhou 450052, China

**Keywords:** PPAR, MAPK pathway, TLR4, esophageal cancer

## Abstract

Although substantial studies on peroxisome proliferator-activated receptor γ (PPARγ) have focused on the mechanisms by which PPARγ regulates glucose and lipid metabolism, recent reports have suggested that PPARγ shows tumorigenic or antitumorigenic effects. The roles and mechanisms of PPARγ activation in esophageal cancer remain unclarified. EC109 and TE10 esophageal cancer cells were treated with 0, 10, 20 and 40 mM of PPARγ agonist rosiglitazone (RGZ) for 24, 48, and 72 h, and the cell viability and apoptosis were detected using methyl thiazolyl tetrazolium (MTT) assay and Flow cytometric (FCM) analysis, respectively. Moreover, the effects of inhibition of PPARγ by antagonist or specific RNA interference on cell viability, apoptosis, the Toll-like receptor 4 (TLR4) and mitogen-activated protein kinase (MAPK) pathways were evaluated. Additionally, the effect of TLR4 signaling on the MAPK pathway, cell viability and apoptosis was assessed. The results showed that RGZ suppressed proliferation and induced apoptosis of esophageal cancer cells, which could be partly restored by inactivation of PPARγ. RGZ suppressed the MAPK and TLR4 pathways, and the inhibitory effect could be counteracted by PPARγ antagonist or specific RNA interference. We also suggested that MAPK activation was regulated by the TLR4 pathway and that blocking the TLR4 and MAPK pathways significantly suppressed proliferation and induced apoptosis of esophageal cancer cells. In conclusion, our data suggested that activation of PPARγ suppressed proliferation and induced apoptosis of esophageal cancer cells by inhibiting TLR4-dependent MAPK pathway.

## INTRODUCTION

Esophageal cancer is an aggressive malignancy, with an estimated 455,800 new esophageal cancer cases and 400,200 deaths worldwide in 2012 [1]. Esophageal cancer is the eighth most common malignancy, and the sixth most common cause of death from cancer worldwide. About 80% of the cases occur in developing countries. It was estimated that about 375,000 Chinese died from esophageal cancer in 2015 [2]. The most common cancer-related complication of esophageal cancer is swallowing difficulty with the cancer development, resulting in malnutrition, pain and lower quality of life [3, 4]. Despite multimodal treatment, long-term survival remains poor with 5-year survival rates of 15-25%, underscoring the urgency to develop novel treatment strategies [5].

Peroxisome proliferator-activated receptor γ (PPARγ) is one of ligand-activated transcription factors within the nuclear steroid hormone receptor superfamily and forms a sub-family along with the other two subtypes, PPARα and PPARβ/δ [6]. Rosiglitazone (RGZ) is known to be a ligand for PPARγ [7]. Transcriptional regulation by PPARs requires heterodimerization with the retinoid X receptor (RXR), and then the heterodimeric complex binds to PPARγ-responsive element (PPRE), consequently triggering the expression of numerous target genes [8, 9]. Although substantial studies on PPARγ have focused on the mechanisms by which PPARγ regulates glucose and lipid metabolism, reports over the past several years have suggested that PPARγ might play additional roles in inflammatory response and cancer. A recent study showed that thiazolidinedione-activated PPARγ had an ability to repress the proliferation of estrogen-dependent breast cancer cells and PPARγ might act as a therapeutic target in human breast cancer [10]. The role of PPARγ in the progression of esophageal cancer remains controversial: activation of PPARγ inhibits cell growth and induces apoptosis *in vitro*; however, PPARγ agonists promote tumor growth in xenografted mice. This discrepancy may be related with *in vivo* effects of “tumor interactions”, PPARγ activation magnitude, and PPARγ-independent effects of agonists [11]. Therefore, the role of PPARγ on esophageal cancer cells and the mechanisms in the response to PPARγ agonists in esophageal cancer cells remain to be further elucidated.

Toll-like receptors (TLRs), expressed on the cell surface, are a group of pattern recognition receptors (PRRs) responsible for recognizing conserved structures unique to bacteria or fungi [12]. Emerging evidence indicates that TLR4 is overexpressed on multiple types of cancer, and plays a crucial role in carcinogenesis, metastasis and cancer development [13], whereas the role of TLRs in esophageal cancer has been studied sparsely [14]. The stimulation of TLR4 with lipopolysaccharide (LPS, a ligand for TLR4) has been revealed to enhance migratory and adhesive properties of esophageal cancer cells [15]. Better understanding of the mechanisms underlying TLR4-dependent tumor formation and progression may be useful for therapy of esophageal cancer.

The signaling components mitogen-activated protein kinases (MAPKs), have key roles in converting external stimuli or environmental stresses into cellular responses [16]. Extracellular signal-related kinase (ERK), c-jun-NH2-terminal kinase (JNK), and p38 MAPK are members of the MAPK signaling pathways [17]. The functions of ERK, JNK and p38 MAPKs in cancer development have been demonstrated [18, 19]. The aim of the present study is to illuminate the signaling network which orchestrates the regulation of TLR4 and MAPK pathway by PPARγ activation in esophageal cancer cells.

## RESULTS

### PPARγ activation suppresses proliferation of esophageal cancer cells

**Figure 1:**
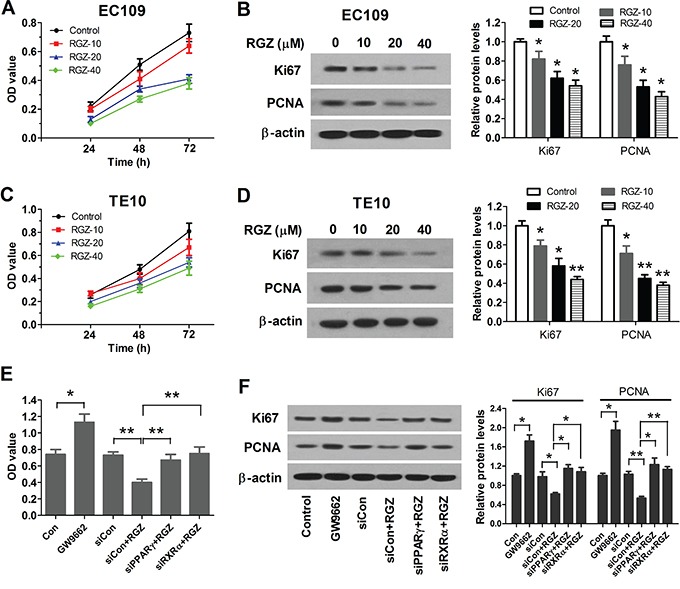
PPARγ activation suppresses proliferation of esophageal cancer cells **A.** RGZ suppresses proliferation of EC109 cells. EC109 cells were treated with 0, 10, 20, and 40 μM of RGZ (Control, RGZ-10, RGZ-20 and RGZ-40) for 24, 48, and 72 h, and proliferation of EC109 cells were determined by using MTT assay. **B.** RGZ decreased the expression levels of Ki67 and PCNA in a dose-dependent manner in EC109 cells. *P < 0.05, compared with the control group. **C.** RGZ suppresses proliferation of TE10 cells. **D.** RGZ decreased the expression levels of Ki67 and PCNA in a dose-dependent manner in TE10 cells. *P < 0.05, **P < 0.01, compared with the control group. **E.** Inhibition of PPARγ activation enhanced proliferation of EC109 cells. EC109 cells were treated with 10 μM of PPAR-γ antagonist GW9662 for 48 h. si-Control or PPARγ- and RXRα-knockdown EC109 cells were treated with 20 μM of PPARγ agonist RGZ for 48 h. *P < 0.05, **P < 0.01. Con: the normal control cells. siCon: si-control cells. **F.** Inactivation of PPARγ inhibits the expression levels of Ki67 and PCNA in EC109 cells. *P < 0.05, **P < 0.01.

Previous studies have proposed that activation of PPARγ by RGZ inhibits growth of various types of cancer [20–22]. To verify the inhibitory effect of activation of PPARγ on esophageal cancer cells, EC109 and TE10 cells were treated with 0, 10, 20, and 40 μM of RGZ for 24, 48, and 72 h, and proliferation of EC109 and TE10 cells were determined using MTT assay. As expected, the proliferation of EC109 and TE10 cells was inhibited by RGZ in time- and dose-dependent manners (Figure 1A and 1C). To confirm the inhibitory effect of activation of PPARγ, EC109 cells were treated with 10 μM of PPAR-γ antagonist GW9662 to test the effect of PPARγ reduction on the proliferation of EC109 cells. We observed that GW9662 enhanced the proliferation of EC109 cells compared with the control group. Moreover, 20 μM of PPARγ agonist RGZ inhibited the proliferation of si-control EC109 cells, but did not repressed the proliferation of PPARγ-knockdown EC109 cells (Figure 1E).

Transcriptional regulation by PPARs requires heterodimerization with RXR [23]. PPARγ and RXRα form a non-symmetric complex, allowing the ligand-binding domain of PPARγ to link multiple domains to both proteins [24]. We observed that siRNA-mediated knockdown of RXRα in EC109 cells blunted the ability of RGZ to repress cell proliferation (Figure 1E). To further confirm the role of PPARγ, western blot analysis was conducted to detect the expression status of Ki67 and PCNA. The expression levels of Ki67 and PCNA were decreased in response to PPARγ activation in EC109 and TE10 cells (Figure 1B and 1D), but increased in the presence of GW9662 or when knockdown of PPARγ or RXRα (Figure 1F). Taken together, all these findings suggest that PPARγ is acting as a heterodimer with RXRα to suppress proliferation of esophageal cancer cells.

### PPARγ activation induces apoptosis of esophageal cancer cells

**Figure 2:**
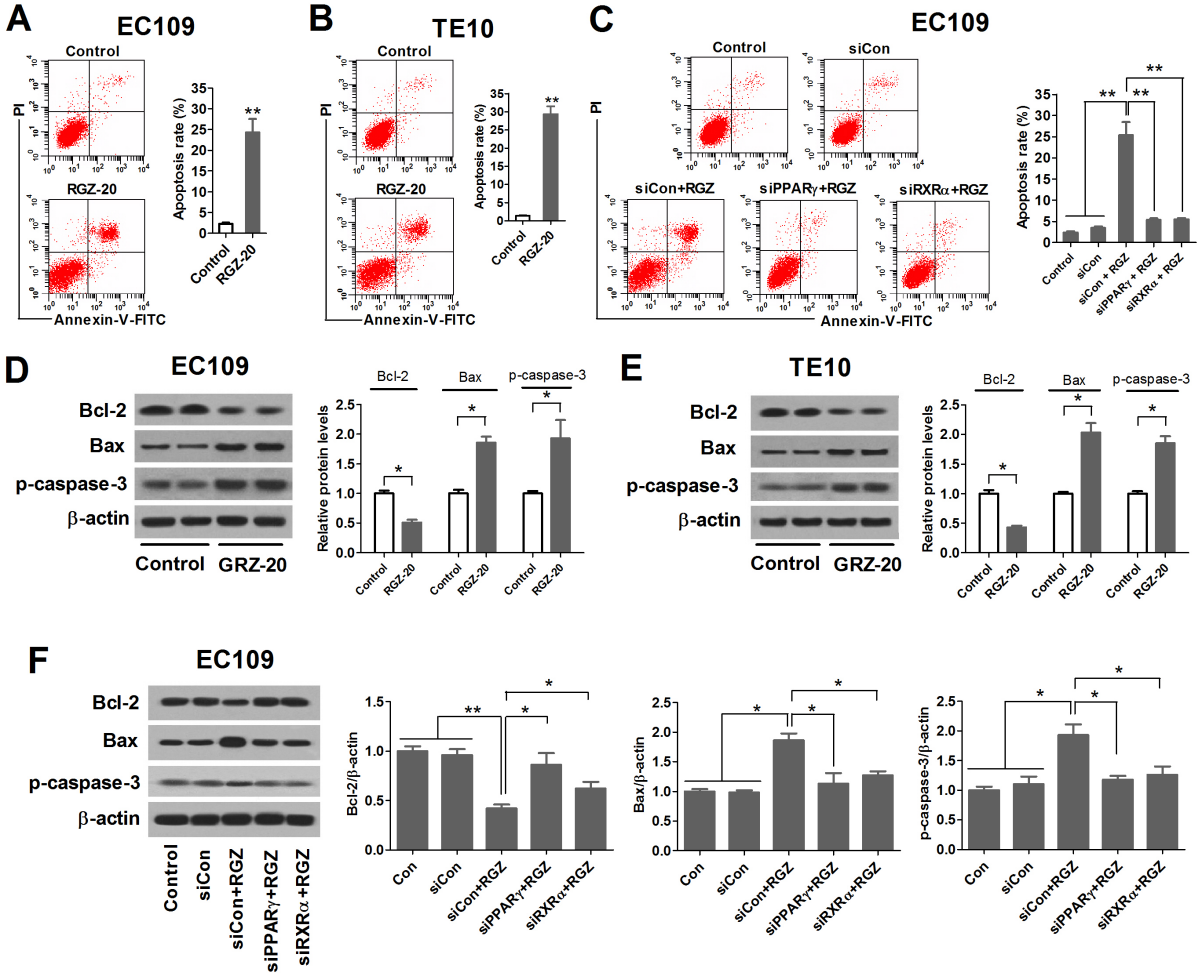
PPARγ activation induces apoptosis of esophageal cancer cells RGZ induces apoptosis of EC109 **A.** and TE10 cells **B.** EC109 and TE10 cells were treated with 20 μM of PPARγ agonist RGZ for 48 h, and apoptosis rate was measured using flow cytometric analysis. **P < 0.01. RGZ-20: cells treated with 20 μM of RGZ. **C.** PPARγ inhibition decreased the apoptosis rates of EC109 cells. Control or PPARγ- and RXRα-knockdown EC109 cells were treated with 20 μM of RGZ for 48 h. **P < 0.01. **D** and **E.** RGZ reduced Bcl-2 expression level, and elevated Bax and cleaved caspase-3 expression levels. *P < 0.05. **F.** The effect of PPARγ inactivation on the expression levels of Bcl-2, Bax and cleaved caspase-3. *P < 0.05, **P < 0.01.

To study the role of PPARγ activation in apoptosis of esophageal cancer cells, EC109 and TE10 cells were treated with 20 μM of PPARγ agonist RGZ for 48 h, and apoptosis rate was measured using flow cytometric analysis. As shown in Figures 2A, 2B, 2D and 2E, RGZ induced apoptosis, reduced Bcl-2 expression level, and elevated Bax and cleaved caspase-3 expression levels in EC109 and TE10 cells. Furthermore, siRNA-mediated knockdown of PPARγ and RXRα in EC109 cells impaired the ability of RGZ to induce apoptosis, reduced Bcl-2 expression level, and elevated Bax and cleaved caspase-3 expression levels (Figure 1C and 1F). These data suggest that PPARγ acts as a heterodimer with RXRα to induce apoptosis of esophageal cancer cells.

### PPARγ agonist inhibits LPS-induced TLR4 activation

**Figure 3:**
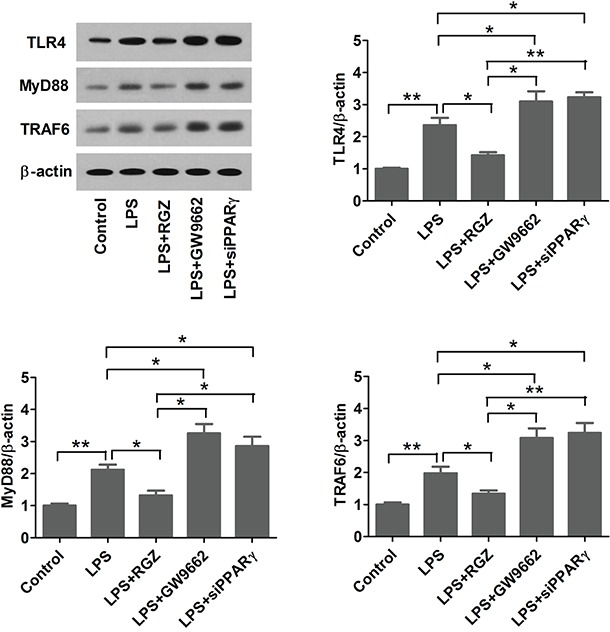
PPARγ agonist inhibits LPS-induced TLR4 activation EC109 cells were pretreated with RGZ (20 μM) or GW9662 (10 μM) overnight, and then stimulated with 10 ng/ml of LPS or vehicle for 1 h. TLR4, MyD88, and TRAF6 expression levels were detected using western bolt analysis. *P < 0.05, **P < 0.01.

A recent study showed that PPARγ agonists attenuated tobacco smoke-induced TLR4 expression in alveolar macrophages [25], and that activated TLR4 has procarcinogenic effects has been demonstrated [26]. MyD88 and TRAF6 are crucial adaptor proteins in TLR4-activated responses [27]. To assess whether PPARγ agonist inhibits LPS-induced TLR4 activation, EC109 cells were pretreated with RGZ (20 μM) or GW9662 (10 μM) overnight, and then stimulated with 10 ng/ml of LPS or vehicle for 1 h. TLR4, MyD88, and TRAF6 expression levels were detected using western bolt analysis. We found that LPS increased the expression levels of TLR4, MyD88, and TRAF6, but the levels were inhibited by RGZ or enhanced by GW9662. Additionally, PPARγ-knockdown cells were stimulated with 10 ng/ml LPS or vehicle for 1 h and TLR4, MyD88, and TRAF6 expression levels were measured using western bolt analysis. The results showed that PPARγ knockdown promoted LPS-induced TLR4, MyD88, and TRAF6 expression in EC109 cells (Figure 3). Collectively, these results indicate that PPARγ agonist inhibits TLR4 signaling in esophageal cancer cells.

### PPARγ agonist inhibits the MAPK pathway in esophageal cancer cells

**Figure 4:**
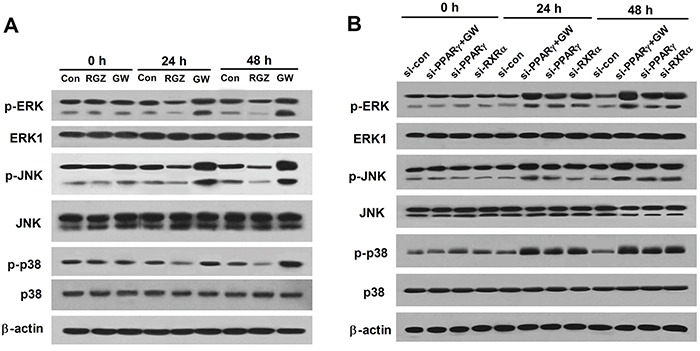
PPARγ agonist inhibits the MAPK pathway in esophageal cancer cells **A.** RGZ decreases the leves of p-ERK, p-JNK, and p-p38, but GW9662 shows the contrary effect. EC109 cells were treated with RGZ (20 μM) or GW9662 (10 μM) for 0, 24, and 48 h, and protein expression levels were detected using western bolt analysis. **B.** PPARγ inhibition increases the leves of p-ERK, p-JNK, and p-p38. PPARγ-knockdown EC109 cells was treated with 10 μM of GW9662 for 0, 24, and 48 h. Western blot analysis was conducted to check the level status of p-ERK, p-JNK, and p-p38 in PPARγ- and RXRα-knockdown EC109 cells.

The activation of ERK, JNK and p38 MAP kinases downstream of TLR4 has been proposed to be involved in the initiation and progression of cancer [28]. To explore whether ERK, JNK and p38 was inhibited by PPARγ activation, EC109 cells were treated with RGZ (20 μM) or GW9662 (10 μM) for 0, 24, and 48 h. As shown in Figure 4A, GW9662 significantly induced p-ERK, p-JNK, and p-p38 activation, but RGZ inhibited the MAPK pathway in EC109 cells. To further validate whether PPARγ was critical for the inactivation of MAPK pathway, western blot analysis was conducted to check the level status of p-ERK, p-JNK, and p-p38 in PPARγ- and RXRα-knockdown EC109 cells. We found that PPARγ- and RXRα-knockdown enhanced the activation of p-ERK, p-JNK, and p-p38. PPARγ-knockdown EC109 cells was treated with 10 μM of GW9662 for 0, 24, and 48 h, the levels of p-ERK, p-JNK, and p-p38 was significantly increased compared to the si-control or si-PPARγ groups (Figure 4B). In summary, these results reveal that PPARγ activation inhibits the MAPK signaling pathway in esophageal cancer cells.

### Inhibition of MAPK pathway by PPARγ agonist requires TLR4

**Figure 5:**
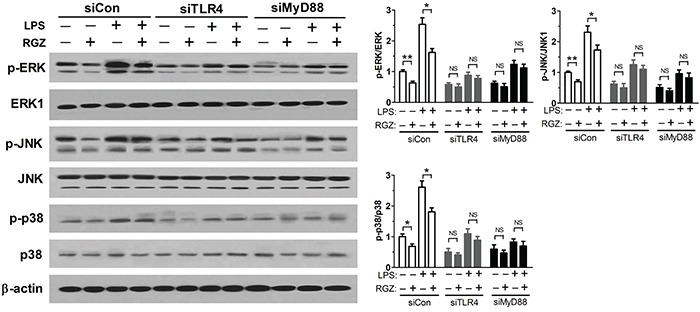
Inhibition of MAPK pathway by PPARγ agonist requires TLR4 TLR4- and MyD88-knockdown EC109 cells were pretreated RGZ (20 μM) overnight, followed by stimulation with 10 ng/ml of LPS or vehicle for 1 h. *P < 0.05, **P < 0.01. NS: not significant.

To examine the ability of PPARγ agonist to inhibit MAPK pathway are dependent on TLR4, TLR4- and MyD88-knockdown EC109 cells were pretreated RGZ (20 μM) overnight, followed by stimulation with 10 ng/ml of LPS or vehicle for 1 h. We found that LPS induced p-ERK, p-JNK, and p-p38 activation, and PPARγ agonist RGZ decreased the expression levels of p-ERK, p-JNK, and p-p38. These inhibitory effects of RGZ were TLR4-dependent, as the inhibitory effects were not observed in TLR4- and MyD88-knockdown cells (Figure 5). These results indicate that inhibition of MAPK pathway by PPARγ agonist requires TLR4.

### Inhibition of TLR4 and MAPK pathways suppresses proliferation and induces apoptosis of esophageal cancer cells

**Figure 6:**
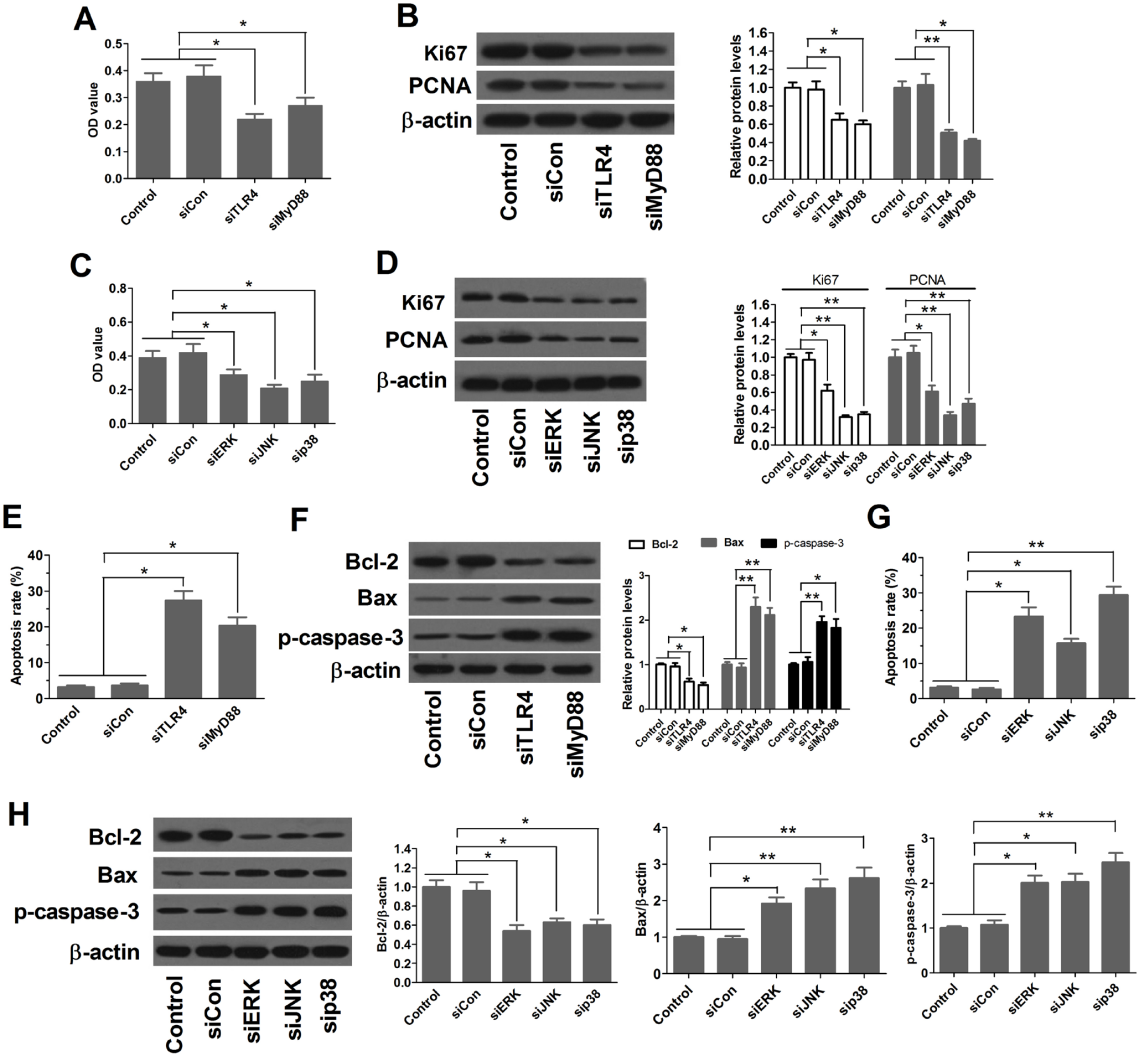
Inactivation of TLR4 and MAPK pathways suppresses proliferation and induces apoptosis of esophageal cancer cells **A** and **B.** Inhibition of TLR4 suppresses proliferation of EC109 cells. The viability and the level status of Ki67 and PCNA were detected using MTT assay and western blot analysis, respectively, 48 h after EC109 cells were transfected with specific siRNAs targeting TLR4, MyD88. *P < 0.05, **P < 0.01. **C** and **D.** Inhibition of MAPK pathways suppresses proliferation of EC109 cells. The viability and the level status of Ki67 and PCNA were detected using MTT assay and western blot analysis, respectively, 48 h after EC109 cells were transfected with specific siRNAs targeting ERK, JNK, and p38. *P < 0.05, **P < 0.01. **E** and **F.** Inhibition of TLR4 induces apoptosis of EC109 cells. The apoptosis rates were determined using FCM analysis 48 h after EC109 cells were transfected with specific siRNAs targeting TLR4, MyD88. *P < 0.05, **P < 0.01. **G** and **H.** Inhibition of MAPK pathways induces apoptosis of EC109 cells. The apoptosis rates were determined using FCM analysis 48 h after EC109 cells were transfected with specific siRNAs targeting ERK, JNK, and p38. *P < 0.05, **P < 0.01.

To address whether inhibition of TLR4 and MAPK pathways was mechanistically linked to the repression of esophageal cancer cells, the viability and apoptosis of EC109 cells transfected with specific siRNAs targeting TLR4, MyD88, ERK, JNK, and p38 were determined using MTT assay and FCM analysis, respectively. Figure 6A and 6C showed that silence of TLR4, MyD88, ERK, JNK, and p38 inhibited proliferation of EC109 cells. Silence of TLR4, MyD88, ERK, JNK, and p38 also suppressed the expression of Ki67 and PCNA (Figure 6B and 6D). As expected, knockdown of TLR4, MyD88, ERK, JNK, and p38 induced apoptosis of EC109 cells (Figure 6E and 6G). Western blot analysis suggested that silence of TLR4, MyD88, ERK, JNK, and p38 decreased Bcl-2 expression level and increased Bax and cleaved caspase-3 expression levels, consistent with the FCM analysis results (Figure 6F and 6H). Taken together, these data suggest that activation of PPARγ inhibits proliferation and induces apoptosis of esophageal cancer cells by inhibiting TLR4-dependent MAPK pathway.

## DISCUSSION

PPARs play a role in the regulation of cancer cell growth. PPARα agonists were recently reported to induce proliferation arrest by negatively regulating cell cycle in pancreatic cancer [29] and reduce primary and metastatic non-small cell lung cancer growth [30]. Zhu *et al*. demonstrated that activation of PPARβ/δ promoted senescence to inhibit tumorigenesis and provided new mechanistic insights into cancer chemoprevention [31]. Though the roles of PPARγ in cancer therapy are debatable, accumulating evidence suggested that activation of PPARγ by agonists exerts an inhibitory effect on cancer cells [32]. For example, PPARγ agonists dramatically reduced cell growth of hepatocellular carcinoma [33], prostate cancer [34], and gastric cancer [35] via regulating the expression and blocking the oncogenic proteins. In our study, we found that PPARγ agonist RGZ had an ability to inhibit proliferation of esophageal cancer cells in time- and dose-dependent manners. Moreover, RGZ induced apoptosis of esophageal cancer cells.

A recent study highlighted that activation of TLR4 by paclitaxel enhances tumor growth and metastasis in breast cancer, and that blocking paclitaxel-induced TLR4 activation in cancer may observably improve therapeutic outcome. [36]. Activation TLR4 by LPS significantly enhanced survival of prostate cancer cells while TLR4 inhibition by a specific inhibitor led to rapid death of prostate cancer cells, [37]. Toll-like receptor 4 (TLR4) has been shown to be upregulated in esophageal squamous cell carcinoma [38]. TLR4 is associated with lymph node metastasis, and stimulation of TLR4 with LPS has been displayed to accelerate migration and adhesion of esophageal squamous cell carcinoma cells [15, 38]. In the present study, we also observed that LPS increased expression levels of TLR4, MyD88, and TRAF6, whereas PPARγ agonist RGZ resisted this effect. We deduced that PPARγ agonist RGZ inhibited progression of esophageal cancer cells via blocking TLR4 pathway in our system. Previous studies demonstrated that PPARγ agonist RGZ suppressed the expression of TLR4 mRNA and protein in alveolar macrophages [25] and that PPARγ agonist pioglitazone attenuated AngiotensinII-induced inflammatory response in cardiac fibroblast cells through inhibition of the TLR4 signaling pathway [39], which are consistent with our study.

The MAPK pathway comprises several crucial signaling components and phosphorylation events, activation of which transmit external signals to modulate cell growth, differentiation, tumorigenesis functions [40]; consequently, the pathway is considered potential therapeutic targets for oncotherapy. Marijn *et al*. showed that miR-634 regulated ERK MAPK and other pathways to affect survival and radiosensitivity of ovarian cancer cells [41]. The response rates of RAF inhibitor monotherapy in BRAF-mutant colorectal cancer are poor, but an ERK inhibitor suppressed MAPK activity and overcame resistance, highlighting the critical dependence of BRAF-mutant colorectal cancers on the MAPK pathway and pointing to potential strategies to overcome clinical resistance [42]. Our data in this study showed that inactivation of MAPK inhibited viability and induced apoptosis of esophageal cancer cells by PPARγ agonist or siRNA targeting ERK, JNK, and p38, which are consistent with these results. PPARγ acted as a molecular switch in moderating myocardial injury via blocking the MAPK pathway in isoproterenol-induced myocardial injury in rats [43]. PPARγ agonist suppressed ERK MAPK signal activation to inhibit activity of cellular NO and reactive oxygen species formation, counteracting LPS-induced inflammatory response in pulp cells [44]. These data suggested that the activation of MAPK pathways is regulated by PPARγ. Moreover, it has been reported that MAPK activation was mediated by TLR4/MyD88 pathway [45]. Ko *et al*. reported that alternaramide, a novel lipophilic depsipeptide, showed anti-inflammatory effects by suppressing activation of TLR4/MyD88-mediated nuclear factor kappa B (NF-кB), JNK and p38 pathways in LPS-stimulated cells [46]. In the present study, we found that ERK, JNK, and p38 MAPK activation was mediated by PPARγ agonist RGZ and the TLR4 pathway. In general, our data suggested that activation of PPARγ suppressed proliferation and induced apoptosis of esophageal cancer cells by inhibiting TLR4-dependent MAPK pathway.

In conclusion, our study demonstrated that PPARγ activation inhibited proliferation and induced apoptosis esophageal cancer cells *in vitro*. PPARγ activation also inactivated the TLR4 and ERK, JNK, and p38 MAPK pathways in esophageal cancer cells. This research highlighted that ERK, JNK, and p38 MAPK activation was regulated by TLR4 pathway, and that inhibition of TLR4, MyD88, ERK, JNK, and p38 attenuated proliferation and induced apoptosis esophageal cancer cells *in vitro*. Therefore, the better understanding of the mechanisms by which PPARγ functions as a suppressor in esophageal cancer is beneficial to PPARγ agonist utilization for the treatment of esophageal cancer.

## MATERIALS AND METHODS

### Cell culture, transfection, and treatment

Human esophageal carcinoma cells EC109 and TE10 cells (ATCC) were purchased from the American Type Culture Collection (Manassas, VA, USA) and maintained in RPMI 1640 medium (Invitrogen, Grand Island, NY, USA) supplemented with 10% fetal bovine serum (Gibco BRL, Carlsbad, CA, USA), 100 U/ml penicillin and 100 μg/ml streptomycin at 37°C in a humidified atmosphere of 5% CO_2_. For RNAi experiments, EC109 and TE10 cells (1 × 10^5^ per well) were seeded in 6-well plates and incubated for 24 h. Cells were transfected with 100 nM specific siRNAs targeting RXRα, PPARγ, TLR4, MyD88, ERK, JNK, and p38 or control siRNA (si-control) with lipofectamine 2000 Reagent (Invitrogen) following the manufacturer's instructions. Cells were harvested at 48 h after incubation and the efficiency of gene knockdown was validated using western blot analysis. Cells treated with different concentrations of RGZ or/and 10 μM of GW9662 were collected to detect the expression levels of Ki67, PCNA, Bcl-2, Bax, and phospho-caspase-3 proteins using western blot analysis. To assess the effects of PPARγ on TLR4 pathway, cells were placed in RPMI 1640 medium containing 10% fetal bovine serum and 20 μM of RGZ, dimethylsulfoxide (DMSO) or 10 μM of GW9662 overnight, and then stimulated with 10 ng/ml lipopolysaccharide (LPS) or vehicle for 1 h. The expression levels of TLR4, MyD88, and TRAF6 were measured using western blot analysis. To assess the effects of PPARγ on MAPK pathway, cells were placed in RPMI 1640 medium containing 10% fetal bovine serum and 20 μM of RGZ or 10 μM of GW9662 for 0, 24, and 48 h, and the expression levels of ERK1, phospho-ERK, JNK, phospho-JNK, p38, and phospho-p38 were measured using western blot analysis.

### MTT assay

Cell viability capacity was assessed using the methyl thiazolyl tetrazolium (MTT) method. EC109 and TE10 cells were plated at a density of 10^4^ per well in 96-well plates with 200 μl medium overnight. After culture media were removed, cells were incubated with 0, 10, 20, and 40 μM of RGZ (Sigma-Aldrich, St Louis, MO, USA) for 0, 24, 48, and 72 h. To assess the effect of GW9662 (Sigma-Aldrich), a PPAR-γ antagonist, on cell viability capacity, EC109 cells were treated with 10 μM of GW9662 for 48 h. For the MTT assay, 50 μl MTT working solution (2.5 mg/ml) was added to each well, followed by continuous incubation for 4 h at 37°C. Culture medium supernatants were removed from each well and 200 μl of dimethyl sulfoxide was added to solubilize the formazan product. The absorbance of each well was measured using a microplate reader at 540 nm. The viability of cells transfected with specific siRNAs targeting RXRα, PPARγ, TLR4, MyD88, ERK, JNK, and p38 or control siRNA was assessed using MTT assay described above. Three independent experiments were conducted in quadruplicate.

### Flow cytometric (FCM) analysis of cell apoptosis

EC109 and TE10 cells were treated with 20 μM RGZ for 48 h, and apoptosis rate was measured using an Annexin V-fluorescein isothiocyanate (FITC)/propidium iodide (PI) Apoptosis Detection Kit (BD PharMingen, San Diego, CA, USA). Cells were collected, and then washed twice with cold PBS and resuspended in 500 μl of Annexin V-FITC binding buffer. The suspension was incubated with 5 μl of Annexin V-FITC and 5 μl of PI at room temperature in the dark for 10 min. Finally, the apoptosis rate was analyzed by fluorescence-activated cell sorting using a BD LSR II flow cytometry kit (BD PharMingen). The apoptosis rates of cells transfected with specific siRNAs targeting RXRα, PPARg, TLR4, MyD88, ERK, JNK, and p38 or control siRNA were measured using FCM analysis described above. Each experiment was conducted for three times independently.

### Western blot analysis

Protein extraction and western blot analysis was performed as described previously [47]. Briefly, proteins were separated by sodium dodecyl sulphate polyacrylamide gel electrophoresis (SDS-PAGE) and transferred to nitrocellulose membranes. After non-specific sites were blocked with 5% non-fat milk, the membranes were incubated with antibodies against Ki67(Abcam, Cambridge, UK), PCNA (Cell Signaling Technology, Danvers, MA, USA), Bcl-2 (Santa Cruz Biotech, Santa Cruz, CA, USA), Bax (Santa Cruz Biotech), phospho-caspase-3 (Santa Cruz Biotech), TLR4 (Sigma-Aldrich), MyD88 (Abcam), TRAF6 (Abcam), ERK1 (Santa Cruz Biotech), phospho-ERK (Thr202/Tyr204; Santa Cruz Biotech), JNK (Santa Cruz Biotech), phospho-JNK (Thr183/Tyr185; Santa Cruz Biotech), p38 (Santa Cruz Biotech), and phospho-p38 (Thr180/Tyr182; Santa Cruz Biotech) and β-actin (Sigma-Aldrich) at 4°C overnight. The membranes were incubated with horseradish peroxidase-conjugated secondary antibodies for 1 h at room temperature, and blots were visualized using the Odyssey Infrared Imaging System (LI-COR Biosciences, Lincoln, NE, USA). β-actin was used as an internal control.

### Statistic analysis

Results are expressed as means ± SE. Statistics were determined using Student's *t* test or one-way analysis of variance (ANOVA). *P* values < 0.05 were considered statistically significant.

## References

[1] Torre LA, Bray F, Siegel RL, Ferlay J, Lortet-Tieulent J, Jemal A (2015). Global cancer statistics, 2012. CA Cancer J Clin.

[2] Chen W, Zheng R, Baade PD, Zhang S, Zeng H, Bray F, Jemal A, Yu XQ, He J (2016). Cancer statistics in China, 2015. CA Cancer J Clin.

[3] Shah MA (2015). Update on metastatic gastric and esophageal cancers. J Clin Oncol.

[4] Cohen DJ, Leichman L (2015). Controversies in the treatment of local and locally advanced gastric and esophageal cancers. J Clin Oncol.

[5] Ebbing EA, Medema JP, Meijer SL, Krishnadath KK, van Berge Henegouwen MI, Bijlsma MF, van Laarhoven HW (2015). HER3 mediates acquired resistance to HER2-targeted therapy in esophageal adenocarcinoma. Cancer Res.

[6] FUJII D, YOSHIDA K, TANABE K, HIHARA J, TOGE T (2004). The ligands of peroxisome proliferator-activated receptor (PPAR) gamma inhibit growth of human esophageal carcinoma cells through induction of apoptosis and cell cycle arrest. Anticancer Res.

[7] Lam M, Samuel C, Royce S, Bourke J (2015). Differential Inhibition Of Airway Contraction By Relaxin And Rosiglitazone. Am J Respir Crit Care Med.

[8] Lemkul JA, Lewis SN, Bassaganya-Riera J, Bevan DR (2015). Phosphorylation of PPARγ Affects the Collective Motions of the PPARγ-RXRα-DNA Complex. PLoS One.

[9] Sato H, Ishihara S, Kawashima K, Moriyama N, Suetsugu H, Kazumori H, Okuyama T, Rumi M, Fukuda R, Nagasue N (2000). Expression of peroxisome proliferator-activated receptor (PPAR) γ in gastric cancer and inhibitory effects of PPARγ agonists. Br J Cancer.

[10] Zhang F, Kong D, Lu Y, Zheng S (2013). Peroxisome proliferator-activated receptor-γ as a therapeutic target for hepatic fibrosis: from bench to bedside. Cell Mol Life Sci.

[11] Fucci A, Colangelo T, Votino C, Pancione M, Sabatino L, Colantuoni V (2012). The role of peroxisome proliferator-activated receptors in the esophageal, gastric, and colorectal cancer. PPAR Res.

[12] Yu H, Ha T, Liu L, Wang X, Gao M, Kelley J, Kao R, Williams D, Li C (2012). Scavenger receptor A (SR-A) is required for LPS-induced TLR4 mediated NF-κB activation in macrophages. Biochim Biophys Acta (BBA)-Mol Cell Res.

[13] Liu W-T, Jing Y-Y, Yu G-f, Han Z-p, Yu D-d, Fan Q-M, Ye F, Li R, Gao L, Zhao Q-D (2015). Toll like receptor 4 facilitates invasion and migration as a cancer stem cell marker in hepatocellular carcinoma. Cancer Lett.

[14] Kauppila JH, Selander KS (2014). Toll-like receptors in esophageal cancer. Front Immunol.

[15] Rousseau MC, Hsu RY, Spicer JD, McDonald B, Chan CH, Perera RM, Giannias B, Chow SC, Rousseau S, Law S (2013). Lipopolysaccharide-induced toll-like receptor 4 signaling enhances the migratory ability of human esophageal cancer cells in a selectin-dependent manner. Surgery.

[16] Wagner EF, Nebreda ÁR (2009). Signal integration by JNK and p38 MAPK pathways in cancer development. Nat Rev Cancer.

[17] Hsu WH, Chen CN, Huang HI, Lai YL, Teng CY, Kuo WH (2012). Urokinase induces stromal cell-derived factor-1 expression in human hepatocellular carcinoma cells. J Cell Physiol.

[18] Wakita A, Motoyama S, Sato Y, Koyota S, Usami S, Yoshino K, Sasaki T, Imai K, Saito H, Minamiya Y (2015). REG Iα activates c-Jun through MAPK pathways to enhance the radiosensitivity of squamous esophageal cancer cells. Tumor Biol.

[19] Sutter AP, Maaser K, Gerst B, Krahn A, Zeitz M, Scherübl H (2004). Enhancement of peripheral benzodiazepine receptor ligand-induced apoptosis and cell cycle arrest of esophageal cancer cells by simultaneous inhibition of MAPK/ERK kinase. Biochem Pharmacol.

[20] Qin L, Gong C, Chen AM, Guo FJ, Xu F, Ren Y, Liao H (2014). Peroxisome proliferator-activated receptor γ agonist rosiglitazone inhibits migration and invasion of prostate cancer cells through inhibition of the CXCR4/CXCL12 axis. Mol Med Rep.

[21] Cerbone A, Toaldo C, Minelli R, Ciamporcero E, Pizzimenti S, Pettazzoni P, Roma G, Dianzani MU, Ullio C, Ferretti C (2012). Rosiglitazone and AS601245 decrease cell adhesion and migration through modulation of specific gene expression in human colon cancer cells. PLoS One.

[22] Zhang H, Jing X, Wu X, Hu J, Zhang X, Wang X, Su P, Li W, Zhou G (2015). Suppression of multidrug resistance by rosiglitazone treatment in human ovarian cancer cells through downregulation of FZD1 and MDR1 genes. Anti-cancer Drugs.

[23] Michalik L, Wahli W (2008). PPARs mediate lipid signaling in inflammation and cancer. PPAR Res.

[24] Chandra V, Huang P, Hamuro Y, Raghuram S, Wang Y, Burris TP, Rastinejad F (2008). Structure of the intact PPAR-γ–RXR-α nuclear receptor complex on DNA. Nature.

[25] Yin Y, Hou G, Li E, Wang Q, Kang J (2014). PPAR Gamma agonists regulate tobacco smoke-induced toll like receptor 4 expression in alveolar macrophages. Respir Res.

[26] Mai CW, Kang YB, Pichika MR (2013). Should a Toll-like receptor 4 (TLR-4) agonist or antagonist be designed to treat cancer? TLR-4: its expression and effects in the ten most common cancers. OncoTargets Ther.

[27] Lin C-C, Lee I-T, Yang Y-L, Lee C-W, Kou YR, Yang C-M (2010). Induction of COX-2/PGE 2/IL-6 is crucial for cigarette smoke extract-induced airway inflammation: Role of TLR4-dependent NADPH oxidase activation. Free Rad Biol Med.

[28] Li W, Fan M, Chen Y, Zhao Q, Song C, Yan Y, Jin Y, Huang Z, Lin C, Wu J (2015). Melatonin Induces Cell Apoptosis in AGS Cells Through the Activation of JNK and P38 MAPK and the Suppression of Nuclear Factor-Kappa B: a Novel Therapeutic Implication for Gastric Cancer. Cell Physiol Biochem.

[29] Hua A, Casper J, Bielenberg D, Schmidt B, Kieran M, Panigrahy D, Gus-Brautbar Y (2015). PPARα: A Novel Target in Pancreatic Cancer. FASEB J.

[30] Skrypnyk N, Chen X, Hu W, Su Y, Mont S, Yang S, Gangadhariah M, Wei S, Falck JR, Jat JL (2014). PPARα Activation Can Help Prevent and Treat Non–Small Cell Lung Cancer. Cancer Res.

[31] Zhu B, Ferry C, Blazanin N, Bility M, Khozoie C, Kang B, Glick A, Gonzalez F, Peters J (2014). PPARβ/δ promotes HRAS-induced senescence and tumor suppression by potentiating p-ERK and repressing p-AKT signaling. Oncogene.

[32] Avena P, Anselmo W, Wang C, Pestell RG, Lamb RS, Casaburi I, Andò S, Martinez-Outschoorn UE, Lisanti MP (2013). Compartment-specific activation of PPARγ governs breast cancer tumor growth, via metabolic reprogramming and symbiosis. Cell Cycle.

[33] Cao L-q, Shao Z-l, Liang H-h, Zhang D-w, Yang X-w, Jiang X-f, Xue P (2015). Activation of peroxisome proliferator-activated receptor-γ (PPARγ) inhibits hepatoma cell growth via downregulation of SEPT2 expression. Cancer Lett.

[34] Bolden A, Bernard L, Jones D, Akinyeke T, Stewart LV (2012). The PPAR gamma agonist troglitazone regulates Erk 1/2 phosphorylation via a PPARγ-independent, MEK-dependent pathway in human prostate cancer cells. PPAR Res.

[35] Cho SJ, Kook MC, Ho Lee J, Shin JY, Park J, Bae YK, Ju Choi I, Won Ryu K, Kim YW (2015). Peroxisome proliferator-activated receptor γ upregulates galectin-9 and predicts prognosis in intestinal-type gastric cancer. Int J Cancer.

[36] Volk-Draper L, Hall K, Griggs C, Rajput S, Kohio P, DeNardo D, Ran S (2014). Paclitaxel therapy promotes breast cancer metastasis in a TLR4-dependent manner. Cancer Res.

[37] Jain S, Suklabaidya S, Das B, Raghav SK, Batra SK, Senapati S (2015). TLR4 activation by lipopolysaccharide confers survival advantage to growth factor deprived prostate cancer cells. Prostate.

[38] Sheyhidin I, Nabi G, Hasim A, Zhang R-P, Ainiwaer J, Ma H, Wang H (2011). Overexpression of TLR3, TLR4, TLR7 and TLR9 in esophageal squamous cell carcinoma. World J Gastroenterol.

[39] Dengfeng G, Meng Z, Liu Z, Dong X (2014). GW25-e5248 PPARγ agonist Pioglitazone Suppresses AngII-induced Inflammation in Cardiac Fibroblast Cells via TLR4-depended Signaling Pathway. J Am Coll Cardiol.

[40] Santarpia L, Lippman SM, El-Naggar AK (2012). Targeting the MAPK-RAS-RAF signaling pathway in cancer therapy. Expert Opin Ther Targets.

[41] van Jaarsveld MT, van Kuijk PF, Boersma AW, Helleman J, van IJcken WF, Mathijssen RH, Pothof J, Berns EM, Verweij J, Wiemer EA (2015). miR-634 restores drug sensitivity in resistant ovarian cancer cells by targeting the Ras-MAPK pathway. Mol Cancer.

[42] Ahronian LG, Sennott EM, Van Allen EM, Wagle N, Kwak EL, Faris JE, Godfrey JT, Nishimura K, Lynch KD, Mermel CH (2015). Clinical acquired resistance to RAF inhibitor combinations in BRAF-mutant colorectal cancer through MAPK pathway alterations. Cancer Discov.

[43] Rani N, Bharti S, Bhatia J, Tomar A, Nag T, Ray R, Arya DS (2015). Inhibition of TGF-β by a novel PPAR-γ agonist, chrysin, salvages β-receptor stimulated myocardial injury in rats through MAPKs-dependent mechanism. Nutrit Metab.

[44] Kim J-C, Lee Y-H, Yu M-K, Lee N-H, Park J-D, Bhattarai G, Yi H-K (2012). Anti-inflammatory mechanism of PPARγ on LPS-induced pulp cells: role of the ROS removal activity. Arch Oral Biol.

[45] Chen I-T, Hsu P-H, Hsu W-C, Chen N-J, Tseng P-H (2015). Polyubiquitination of Transforming Growth Factor β-activated Kinase 1 (TAK1) at Lysine 562 Residue Regulates TLR4-mediated JNK and p38 MAPK Activation. Sci Rep.

[46] Ko W, Sohn JH, Jang J-H, Ahn JS, Kang DG, Lee HS, Kim J-S, Kim Y-C, Oh H (2016). Inhibitory effects of alternaramide on inflammatory mediator expression through TLR4-MyD88-mediated inhibition of NF-кB and MAPK pathway signaling in lipopolysaccharide-stimulated RAW264. 7 and BV2 cells. Chem Biol Interac.

[47] Wu K, Yang Y, Zhao J, Zhao S (2016). BAG3-mediated miRNA let-7g and let-7i inhibit proliferation and enhance apoptosis of human esophageal carcinoma cells by targeting the drug transporter ABCC10. Cancer Lett.

